# Seven protective miRNA signatures for prognosis of cervical cancer

**DOI:** 10.18632/oncotarget.10678

**Published:** 2016-07-18

**Authors:** Bei Liu, Jin-feng Ding, Jian Luo, Li Lu, Fen Yang, Xiao-dong Tan

**Affiliations:** ^1^ School of Public Health, Wuhan University, Wuhan, 430071, China; ^2^ Department of Anesthesiology, Taizhou Hospital, Taizhou, 317000, China; ^3^ Department of Geriatric Medicine, Tongji Hospital, Huazhong University of Science and Technology, Wuhan, 430030, China; ^4^ Cancer Epigenetics Laboratory, Department of Clinical Oncology, Sir YK Pao Center for Cancer and Li Ka Shing Institute of Health Science, Chinese University of Hong Kong, Hong Kong, 999077, China; ^5^ Department of Nursing, Hubei University of Chinese Medicine, Wuhan, 430063, China

**Keywords:** cervical cancer, survival analysis, Cox's model, GO enrichment, pathway analysis

## Abstract

Cervical cancer is the second cause of cancer death in females in their 20s and 30s, but there were limited studies about its prognosis. This study aims to identify miRNA related to prognosis and study their functions. TCGA data of patients with cervical cancer were used to build univariate Cox's model with single clinical parameter or miRNA expression level. Multivariate Cox's model was built using both clinical information and miRNA expression levels. At last, STRING was used to enrich gene ontology or pathway for validated targets of significant miRNAs, and visualize the interactions among them. Using univariate Cox's model with clinical parameters, we found that two clinical parameters, tobacco use and clinical stage, and seven miRNAs were highly correlated with the survival status. Only using the expression level of miRNA signatures, the model could separate patients into high-risk and low-risk groups successfully. An optimal feature-selected model was proposed based on two clinical parameters and seven miRNAs. Functional analysis of these seven miRNAs showed they were associated to various pathways related to cancer, including MAPK, VEGF and P53 pathways. These results helped the research of identifying targets for targeted therapy which could potentially allow tailoring of treatment for cervical cancer patients.

## INTRODUCTION

Cervical cancer is the second cause of cancer death in females in their 20s and 30s. It was estimated that there would be 12,900 new cases and 4,100 death in the United States in 2015 [1]. Patients with cervical cancer may have significantly different clinical outcomes, which are hard to predict. If the group of high-risk patients were identified, they could got modified treatment including higher radiation doses. As a result, however, it is difficult to apply personalized treatment to patients with cervical cancer due to the fact we cannot predict the outcome using prediction models to date, which may improve clinical outcomes significantly.

The expression level of miRNA have been considered as potential biomarkers for predicting survival status for various human cancers [2]. However, there were few studies that investigated the prognostic value of miRNAs in cervical cancer. Only Hu et al [3] found two miRNA biomarkers for cervical cancer prognosis with a smaller size of patients, but other studies [4] focused on specific miRNAs and cell line experiments instead of real patients survival status. Given the fact that the poor survival for patients with advanced stages of cervical cancer, here we aim to identify miRNA which can predict the prognosis of cervical cancer and study their functions by analyzing their targeted mRNAs, which may be used to predict the survival time and help determine the therapy in the future.

## RESULTS

### Clinical parameters related to prognosis

After filtering, 166 censored and 48 non-censored patients were used in the following analysis. Their clinical characteristics were shown in Table 1. Selected clinical parameters were utilized to fit the Cox's model Table 2. Parameters which were unavailable in many patients, like whether lymphovascular was involved or whether the patient was infected with HPV, were not included, even though lymphovascular involvement had a significant p-value and HPV infection is an important cause of cervical cancer. Among these clinical characteristics, only tobacco use and clinical stage had a p-value less than 0.05. On the other hand, tumor grade or histologic diagnosis was not significant.

**Table 1:** Clinical characteristics of 214 cervical cancer patients after filtering

Characteristic	Censored (n=166)	Non-Censored (n=48)
Age (Mean ± SD)	47.2 ± 13.1	52.5 ± 16.2
Tumor Grade		
Gx	16	6
G1	11	2
G2	69	23
G3	68	17
G4	1	0
Unknown	1	0
Clinical Stage		
Stage I	92	21
Stage II	44	8
Stage III	20	8
Stage IV	5	11
Unknown	5	0
Histology		
Squamous	135	40
Adenocarcinoma	31	8

**Table 2:** Univariate Cox analysis of clinical parameters with the prognosis

Clinical variable	P Value^$^
Ethnicity	5.80E-01
Tumor grade	5.42E-01
Histologic diagnosis	2.71E-01
Race	2.12E-01
Age at diagnosis	9.44E-02
Tobacco use^#^^%^	4.49E-02
Clinical stage^&^^%^	2.61E-04

$ P-values were obtained from Likelihood ratio test; patients omitted when data is unavailable

# Patients were separated into three groups; that is, current smoker, non-smoker and current reformed smoker

& Clinical stages were simplified into four stage; that is, I, II, III and IV. They were treated as numeric.

% Significant variables had a p-value less than 0.05

### miRNA signature related to prognosis

Seven miRNAs had an FDR less than 0.05, which were regarded as significantly correlated with survival time (Table 3). All of these miRNAs have protective impacts on the survival time based on relationship between their expression and survival status; that is, the high expression of these miRNAs leads to a longer survival time.

**Table 3:** Seven miRNA signature correlated with prognosis

No	miRNA	P Value^$^	FDR	Type^#^
1	hsa-mir-142	6.27E-06	1.96E-03	Protective
2	hsa-mir-642a	8.25E-06	1.96E-03	Protective
3	hsa-mir-101-1	7.82E-05	1.18E-02	Protective
4	hsa-mir-3607	9.93E-05	1.18E-02	Protective
5	hsa-mir-502	1.45E-04	1.38E-02	Protective
6	hsa-mir-378c	2.31E-04	1.83E-02	Protective
7	hsa-mir-150	4.33E-04	2.93E-02	Protective

$ P-values were obtained from Likelihood ratio test in univariate Cox's model

# Types included protective and risky, but no risky miRNA was found here

### Performance of miRNA signature

**Figure 1:**
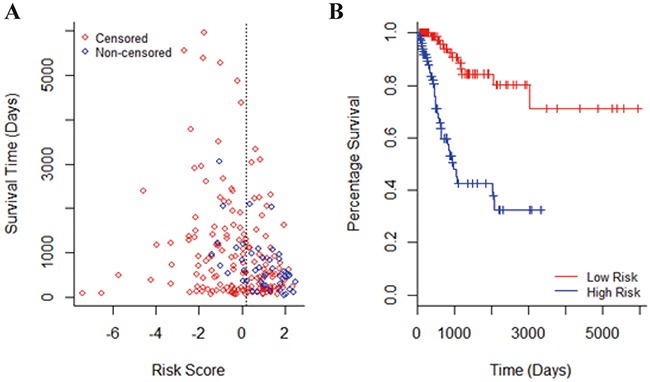
The performance of seven miRNA signature **A.** Survival status of patients along with corresponding risk score. The blue dots represented patients who died and the red dots represented censored patients. Patients were separated into two groups by their median which was shown by the dotted line. Patients in the low-risk group were on the left side of the line and patients with high risk were on the right. **B.** The Kaplan-Meier curves estimate overall survival of cervical cancer patients according to the seven miRNA signature. The p-value of log-likelihood test for differences between the low-risk and high-risk groups was 1.13E-08.

After building the multivariate Cox's model, the hazard ratio was assigned to each patients. The comparison of hazard ratio with patient survival time was shown in Figure 1A. Patients were divided into two groups evenly. Most of patients who died of cancer and survived for a relatively shorter time were located on the right side of the cutoff. To ensure that the selected miRNA signature was able to separate patients into low-risk and high-risk groups, the Kaplan-Meier curve was plotted using the group separation of hazard ratio (Figure 1B). The p-value, representing the separation of two groups, was only 1.13E-08 by log-likelihood test.

**Figure 2:**
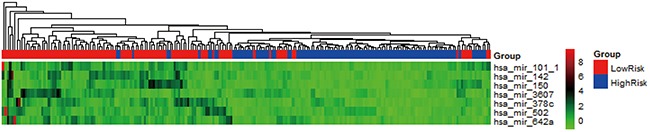
Heat map of seven miRNA signature of patients The rows represented miRNA signature and columns represent patients. The assigned low-risk group and high-risk group according to hazard ratio were shown on the top of the heat map.

The heat map (Figure 2) showed that when the patients were clustered using scaled expression level of seven miRNA signatures, these miRNAs was able to separate the patients into low-risk and high-risk group, which indicated that the high expression of these miRNAs led to a lower hazard ratio and a longer survival time.

### Multivariate model with clinical parameters

With seven miRNAs and two clinical parameters, the multivariate model only had two variables having a p-value lower than 0.05. As a result, after building the multivariate model, a feature-selected procedure was took. The final model contains miR-150, miR-3607, miR-378c, miR-642a, smoking status and clinical stage. Due to the amount of variables in the original model, we believed this final model was more suitable to use in clinics. Considering that the arbitrary coefficients change with different methods or kinds of microarray used, they had no value to be shown here. However, we proposed to utilize the expression levels of four miRNAs and two clinical characteristics as signature for predicting prognosis of cervical cancer, which should be tested in the wet lab or in clinics.

After splitting patients into the train group and test group randomly, the final model highly depended on how the patients were separated. The main reason is the limited non-censored patients (only 48) and the within-group variations. To test the final model, other independent data set is needed.

### Validated mRNA targets

Nowadays, tools for predicting mRNA targets of miRNAs, which may cause high false positive and false negative rate, are still immature. As a result, we used validated targets of miRNA from miRTarBase [5] to finish the following analysis (Supplementary Table S1). Still, considering the high false positive hit of NGS data, only mRNAs with strong evidences were left. Some of miRNAs didn't have any mRNA targets with strong evidences, so they were neglected here. The validated miRNA-mRNA interactions were shown in Table 4.

**Table 4:** Mature sequences of miRNA signature and experimentally validated mRNA targets

Mature miRNA	Targets	Mature miRNA	Targets
hsa-miR-101-3p	APP	hsa-miR-101-5p	ATM
	ARID1A		PRKDC
	ATM		
	ATP5B	hsa-miR-142-3p	ARNTL
	ATXN1		PROM1
	DNMT3A		RAC1
	DUSP1		
	EED	hsa-miR-150-5p	CXCR4
	EZH2		EGR2
	FBN2		EP300
	FMR1		FLT3
	FOS		IGF2
	MCL1		MYB
	MEIS1		NOTCH3
	MYCN		P2RX7
	PTGS2		TP53
	SOX9		VEGFA
	STMN1		
	SUZ12	hsa-miR-642a-5p	ZEB1
			DOHH
hsa-miR-142-5p	NFE2L2		

### Gene ontology and pathway enrichment

Gene ontology and pathway enrichment using online tool STRING [6]. The gene ontology included here are just slim gene ontology, which gives an overview without the details of the specific terms [7]. Interestingly, the enriched gene ontology seemed more related to embryo development than cancer. In the category of biological processes, only homeostatic process had close relationship with the cancer progression. On the other hand, other three terms were all related to structure development, which might be induced by the fact that these miRNAs were obtained from cancer in cervix, which in turn bind to mRNA highly expressed in cervix (Table 5).

**Table 5:** Slim gene ontology enrichment

Category	ID	Term	P Value	FDR
Biological processes	0048646	anatomical structure formation	3.03E-11	4.08E-07
		involved in morphogenesis		
	0009790	embryo development	1.52E-08	1.02E-04
	0048856	anatomical structure	4.53E-08	2.03E-04
		development		
Cellular components	0042592	homeostatic process	2.56E-07	8.62E-04
	0043234	protein complex	3.73E-07	5.91E-04
	0005654	nucleoplasm	3.93E-06	3.11E-03

Most of the enriched KEGG pathways were associated with cancer, which supported our predicted miRNAs that they played important roles in the cancer development and progression (Table 6). Six out of eight enriched pathways were involved in cancer directly, except that two pathways were associated with virus infection. Supplementary Table S2 contained more enriched KEGG pathways which had adjusted p-value with method Bonferroni less than 0.05. Most of the additional pathways were also associated with cancer or virus infection.

**Table 6:** KEGG pathway enrichment

ID	Term	p-value	FDR
5206	MicroRNAs in cancer	6.82E-16	1.96E-13
5202	Transcriptional misregulation in cancer	1.28E-08	1.84E-06
5200	Pathways in cancer	1.21E-06	1.16E-04
5166	HTLV-I infection	4.49E-06	3.22E-04
4110	Cell cycle	5.93E-05	3.40E-03
4010	MAPK signaling pathway	7.38E-05	3.53E-03
5161	Hepatitis B	1.02E-04	4.16E-03
5210	Colorectal cancer	1.37E-04	4.82E-03
4370	VEGF signaling pathway	1.52E-04	4.82E-03
5212	Pancreatic cancer	1.68E-04	4.82E-03

### Network analysis of targeted miRNAs

**Figure 3:**
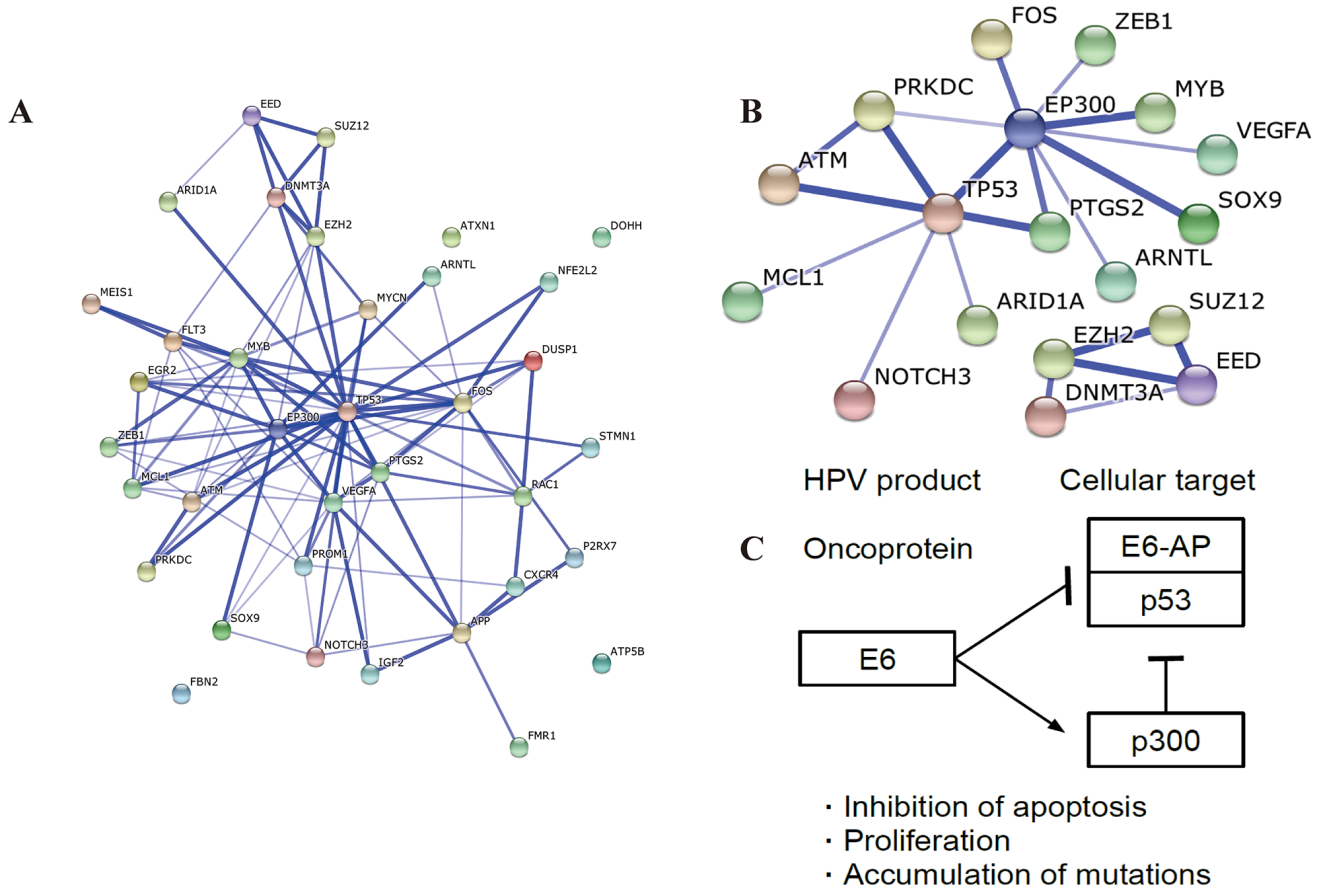
Network of proteins **A.** The network predicted with methods of neighborhood, gene fusion, co-occurrence, co-expression, experiments, databases and text-mining. This network was enriched in interaction with a p-value of 5.55E-15. **B.** The network predicted only with experiments. Disconnected nodes were hidden. This network was not enriched in interaction. **C.** KEGG pathway related to cervical cancer. Only the part related to results of this study is plotted. This graph was reorganized based on viral carcinogenesis (hsa05203) from KEGG.

STRING [6] was used to visualize the protein interaction. Figure 3A should the network with methods of neighborhood, gene fusion, co-occurrence, co-expression, experiments, databases and text-mining. This network was enriched in interaction, which assumed that these protein worked altogether and joined in the related pathways. After removing evidences with lower confidence and only using data experiments, a network with fewer genes was built (Figure 3B). In both networks, TP53 and EP300 were both the centers and had high degrees, indicating the important roles of them in the prognosis of cervical cancer. In fact, both of TP53 and EP300 are the targets of human papillomavirus (HPV), which is the top risk factor of cervical cancer.

## DISCUSSION

In this study, we identified two clinical parameters and seven miRNAs as biomarkers for the prognosis of cervical cancer. All of these miRNAs are shown to have protective impacts on the survival time in this study, whose high expression leads to a longer survival time.

Compared with results of Hu, Schwarz [3], they found that only the number of lymph node involved in cervical cancer was negatively associated with the disease outcome. In this study, lymph node was also significant after removing missing data. However, we found that clinical stage and tobacco use were both highly correlated with survival time. In fact, tobacco use is an important risk factor for cervical cancer, which was ignored in the study of Hu, Schwarz [3].

It was reported that miR-142 worked as a cancer suppressor in colon cancer [8]. On the meantime, it can be used as one part of prognosis predictor in gastric cancer and esophageal squamous cell cancer [9, 10]. Few studies between miR-642a and cancer were found. For miR-101, it was proposed that the loss of it lead to overexpression of EZH2 and cancer progression in further [11, 12]. miR-502 inhibited colon cancer growth by a negative feedback loop with p53 [13] and was correlated with early development of breast cancer [14]. miR-378c was downregulated in the rectal cancer and gastric cancer [15, 16] but its expression may enhance cell survival and tumor growth [17]. This result fought against its protective role in this study. miR-510 was suggested to be a potential biomarker of colorectal cancer and lower expression suggested a shorter survival time [18]. However, Wu, Jin [19] reported that overexpression of miR-150 resulted in proliferation and growth of gastric cancer. Such contradiction may come from the fact that each miRNA has dozens of targets and iteither promotes or inhibits the cancer through different pathways based on the cancer types and specific cellular environment.

Most of the seven miRNA predictors were reported to play roles in the cancer, which strengthened the possibility to use the seven miRNA signature as predictor of prognosis of cervical cancer. The mRNAs regulated by miRNA signatures were enriched in pathways which are involved in cancer progression orprognosis. The most significant pathway enriched was microRNAs in cancer, which was certainly accordance with analysis procedures. The results suggested that our obtained seven miRNA signature were in fact highly correlated with cancer.

Pathways of colorectal cancer and pancreatic cancer were enriched, while no cervical cancer pathway was obtained since the specific pathway of cervical cancer is unavailable in KEGG. MAPK and VEGF signaling pathway were enriched, which are critically important pathways in cancer development. It was also reported that oncoprotein E5 activated the MAPK pathway and helped the activities of E6 and E7 [20]. Another stuff should be noticed is the HTLV-I infection and Hepatitis B pathway. Infection of both viruses may lead to cancer, and involve MAPK and P53 signaling pathway [21]. Interestingly, oncoprotein E6 of HPV targets TP53 and EP300 and results in inhibition of apoptosis, cancer proliferation and accumulations of mutations shown in Figure 3C [21]. When it comes to the results of protein network, TP53 and EP300 were both the centers of the network [22]. Moreover, due to the amount of variables in the original model, we believed our final model was more suitable to use in clinics. Considering that the arbitrary coefficients change with different methods or kinds of microarray used, they had no value to be shown here. However, we proposed to utilize the expression levels of four miRNAs and two clinical characteristics as signature for predicting prognosis of cervical cancer, which should be tested in the wet lab or in clinics.

To summary, our study figured out seven miRNAs which had protective impacts on the survival status of cervical cancer patients, which suggested that these miRNAs played a critical role in the pathogenesis, progression and prognosis of cervical cancer. Patients with a higher hazard ratio had worse survival status in the Cox's model. These miRNAs play their role by binding to mRNAs enriched in various pathways related to cancer. mRNAs may affect the prognosis of cervical cancer through MAPK, P53 and VEGF pathway. These results helped the research of identifying targets of targeted therapy. The only drug of targeted therapy now used in advanced cervical cancer is bevacizumab and targets the VEGF signaling pathway [23]. This study suggested that drugs targeted the MAPK and P53 pathways may also work.

## MATERIALS AND METHODS

### TCGA cervical cancer dataset

Expression levels of miRNA and clinical information of patients with cervical squamous cell carcinoma and endocervical adenocarcinoma (CESC) were retrieved from The Cancer Genome Atlas (TCGA). Level 3 data of miRNA expression level, including 1046 miRNAs, were downloaded. The dataset contained 307 patients with solid tumor, 2 patients with metastatic tumor and 3 samples from normal solid tissues. Only patients with solid tumor were used in this analysis considering the limited size of samples with metastatic tumor. The clinical information and follow-up data of these patients were also obtained to extract the information of recurrence time.

Patients were filtered by the following criteria. First, patients who did not have tumor recurrence but died were removed. Second, the cases that patients were alive and the last contact days were unavailable were discarded. Third, patients with less than 50-day follow-up were removed, which were all censored data.

### Univariate survival analysis

Clinical information was used to build univariate Cox proportional hazard ratio model [24], including clinical stage, tumor grade and so forth. If one parameter was unavailable in certain patients, these patients were treated as missing. Significant parameters were filtered out using 0.05 as the cutoff.

Each miRNA was fitted to univariate Cox's model as well [24]. FDR adjustment was carried out on the results of miRNA expression data and significant miRNAs were selected with a cutoff of 0.05. Seven miRNAs were identified as biomarker of prognosis.

Next, whether these miRNAs can really separate patients into high-risk and low-risk groups was checked. A multivariate Cox's model was built based on significant miRNA expression data first and patients were separated into two groups using proportional hazard ratio [25]. Median of the all hazard ratios was used as a cutoff to evenly separate the patients in this case. The log-rank test [26] was carried out to get the quantitative measure (p-value) of the separation of the two groups and Kaplan-Meier curves [27] was plot to visualize such separation. Moreover, using scaled expression level of these seven miRNAs, a heat map was plotted with the annotation of high-risk or low-risk group to visualize the relationship between expression level and groups.

### Multivariate survival analysis

On the meantime, the clinical information was considered to be significant and was included into the Cox's model. Seven miRNAs, together with two clinical characteristics, were used to build the model. After that, an optimal formula was constructed using Akaike information criterion (AIC) based on the log-likelihood ratio [28] from this model, which was believed to be a better model.

### GO and pathway of targets of miRNAs

Validated targets of miRNAs were available in miRTarBase [5]. Only miRNA-target interactions with strong evidences, either by reporter assay or western blot, were extracted. Gene ontology enrichment [7] and pathway enrichment [21] were carried out based on these validated targets using online tool STRING [6]. The interactions among products of these targets were visualized by the same tool.

## SUPPLEMENTARY MATERIAL TABLES







## References

[1] Siegel RL, Miller KD, Jemal A (2015). Cancer statistics 2015. CA Cancer J Clin.

[2] Jay C, Nemunaitis J, Chen P, Fulgham P, Tong AW (2007). miRNA profiling for diagnosis and prognosis of human cancer. DNA Cell Biol.

[3] Hu X, Schwarz JK, Lewis JS, Huettner PC, Rader JS, Deasy JO, Grigsby PW, Wang X (2010). A microRNA expression signature for cervical cancer prognosis. Cancer research.

[4] Pang RT, Leung CO, Ye TM, Liu W, Chiu PC, Lam KK, Lee KF, Yeung WS (2010). MicroRNA-34a suppresses invasion through downregulation of Notch1 and Jagged1 in cervical carcinoma and choriocarcinoma cells. Carcinogenesis.

[5] Hsu SD, Tseng YT, Shrestha S, Lin YL, Khaleel A, Chou CH, Chu CF, Huang HY, Lin CM, Ho SY, Jian TY, Lin FM, Chang TH, Weng SL, Liao KW, Liao IE (2014). miRTarBase update 2014: an information resource for experimentally validated miRNA-target interactions. Nucleic Acids Res.

[6] Szklarczyk D, Franceschini A, Wyder S, Forslund K, Heller D, Huerta-Cepas J, Simonovic M, Roth A, Santos A, Tsafou KP, Kuhn M, Bork P, Jensen LJ, von Mering C (2015). STRING v10: protein-protein interaction networks, integrated over the tree of life. Nucleic Acids Res.

[7] Gene Ontology C (2015). Gene Ontology Consortium: going forward. Nucleic Acids Res.

[8] Shen WW, Zeng Z, Zhu WX, Fu GH (2013). MiR-142-3p functions as a tumor suppressor by targeting CD133 ABCG2 and Lgr5 in colon cancer cells. J Mol Med (Berl).

[9] Zhang X, Yan Z, Zhang J, Gong L, Li W, Cui J, Liu Y, Gao Z, Li J, Shen L, Lu Y (2011). Combination of hsa-miR-375 and hsa-miR-142-5p as a predictor for recurrence risk in gastric cancer patients following surgical resection. Ann Oncol.

[10] Lin RJ, Xiao DW, Liao LD, Chen T, Xie ZF, Huang WZ, Wang WS, Jiang TF, Wu BL, Li EM, Xu LY (2012). MiR-142-3p as a potential prognostic biomarker for esophageal squamous cell carcinoma. J Surg Oncol.

[11] Varambally S, Cao Q, Mani RS, Shankar S, Wang X, Ateeq B, Laxman B, Cao X, Jing X, Ramnarayanan K, Brenner JC, Yu J, Kim JH, Han B, Tan P, Kumar-Sinha C (2008). Genomic loss of microRNA-101 leads to overexpression of histone methyltransferase EZH2 in cancer. Science.

[12] Smits M, Nilsson J, Mir SE, van der Stoop PM, Hulleman E, Niers JM, de Witt Hamer PC, Marquez VE, Cloos J, Krichevsky AM, Noske DP, Tannous BA, Wurdinger T (2010). miR-101 is down-regulated in glioblastoma resulting in EZH2-induced proliferation migration and angiogenesis. Oncotarget.

[13] Zhai H, Song B, Xu X, Zhu W, Ju J (2013). Inhibition of autophagy and tumor growth in colon cancer by miR-502. Oncogene.

[14] Song F, Zheng H, Liu B, Wei S, Dai H, Zhang L, Calin GA, Hao X, Wei Q, Zhang W, Chen K (2009). An miR-502-binding site single-nucleotide polymorphism in the 3′-untranslated region of the SET8 gene is associated with early age of breast cancer onset. Clin Cancer Res.

[15] Li X, Zhang G, Luo F, Ruan J, Huang D, Feng D, Xiao D, Zeng Z, Chen X, Wu W (2012). Identification of aberrantly expressed miRNAs in rectal cancer. Oncol Rep.

[16] Xie J, Chen M, Zhou J, Mo MS, Zhu LH, Liu YP, Gui QJ, Zhang L, Li GQ (2014). miR-7 inhibits the invasion and metastasis of gastric cancer cells by suppressing epidermal growth factor receptor expression. Oncol Rep.

[17] Lee DY, Deng Z, Wang CH, Yang BB (2007). MicroRNA-378 promotes cell survival tumor growth and angiogenesis by targeting SuFu and Fus-1 expression. Proc Natl Acad Sci U S A.

[18] Ma Y, Zhang P, Wang F, Zhang H, Yang J, Peng J, Liu W, Qin H (2012). miR-150 as a potential biomarker associated with prognosis and therapeutic outcome in colorectal cancer. Gut.

[19] Wu Q, Jin H, Yang Z, Luo G, Lu Y, Li K, Ren G, Su T, Pan Y, Feng B, Xue Z, Wang X, Fan D (2010). MiR-150 promotes gastric cancer proliferation by negatively regulating the pro-apoptotic gene EGR2. Biochem Biophys Res Commun.

[20] Moody CA, Laimins LA (2010). Human papillomavirus oncoproteins: pathways to transformation. Nat Rev Cancer.

[21] Kanehisa M, Goto S, Sato Y, Kawashima M, Furumichi M, Tanabe M (2014). Data information knowledge and principle: back to metabolism in KEGG. Nucleic Acids Res.

[22] Munoz N, Castellsague X, de Gonzalez AB, Gissmann L (2006). Chapter 1: HPV in the etiology of human cancer. Vaccine.

[23] Zagouri F, Sergentanis TN, Chrysikos D, Filipits M, Bartsch R (2012). Molecularly targeted therapies in cervical cancer. A systematic review. Gynecol Oncol.

[24] Andersen PK, Gill RD (1982). Cox's regression model for counting processes: a large sample study. Ann Stat.

[25] Lossos IS, Czerwinski DK, Alizadeh AA, Wechser MA, Tibshirani R, Botstein D, Levy R (2004). Prediction of survival in diffuse large-B-cell lymphoma based on the expression of six genes. N Engl J Med.

[26] Mantel N (1966). Evaluation of survival data and two new rank order statistics arising in its consideration. Cancer Chemother Rep.

[27] Kaplan EL, Meier P (1958). Nonparametric Estimation from Incomplete Observations. Journal of the American Statistical Association.

[28] deLeeuw J, Kotz S, Johnson NL (1992). Introduction to Akaike (1973) information theory and an extension of the maximum likelihood principle. Breakthroughs in Statistics Volume 1.

