# Supplementary material for: Seven protective miRNA signatures for prognosis of cervical cancer
Source: Oncotarget. 2016 Jul 18;7(35):56690–8. doi: 10.18632/oncotarget.10678 (PMC5302945; doi:10.18632/oncotarget.10678)
Supplement: Supplementary file 1 [file oncotarget-07-56690-s001.pdf]

Seven protective miRNA signatures for prognosis of cervical cancer

SUPPLEMENTARY TABLES

Supplementary Table S1:

See Supplementary File 1

Supplementary Table S2:

See Supplementary File 2
